# High prevalence of hepatitis E virus infection among domestic pigs in Ibaraki Prefecture, Japan

**DOI:** 10.1186/s12917-019-1816-x

**Published:** 2019-03-12

**Authors:** Takumi Motoya, Masahiro Umezawa, Keiko Goto, Ikuko Doi, Noriko Nagata, Yoshiaki Ikeda, Atsushi Sakuta, Nobuya Sasaki, Koji Ishii

**Affiliations:** 1 Ibaraki Prefectural Institute of Public Health, Mito, Ibaraki 310-0852 Japan; 2 Laboratory of Laboratory Animal Science and Medicine, Faculty of Veterinary Medicine, Kitasato University, Towada, Aomori Japan; 3 Swine Laboratory, Ibaraki Prefectural Livestock Research Center, Inashiki, Ibaraki Japan; 4 Department of Virology II, National Institute of Infectious Diseases, Musashimurayama, Tokyo Japan

**Keywords:** Hepatitis E virus, Ibaraki prefecture, Piglets, Slaughterhouse

## Abstract

**Background:**

Hepatitis E virus (HEV) is prevalent in pigs and may serve as a reservoir for human infection. However, data on HEV infections in pigs in Ibaraki Prefecture, Japan, are limited. Here, we clarified the process and course of HEV in naturally infected pigs. Serum (*n* = 160) and liver (*n* = 110) samples were collected from pigs at the slaughterhouse. Furthermore, serum samples were collected from 45 breeding sows and serum and feces samples were collected from 7 piglets once a week (raised until 166 days of age). HEV antigen and antibodies were evaluated, and the genotype was identified based on molecular phylogenetic tree analysis.

**Results:**

The samples collected from the slaughterhouse revealed that few pigs were HEV carriers but most possessed anti-HEV antibodies. Most breeding sows possessed antibodies, and the piglets excreted HEV on the farm at approximately 10 weeks of age. One pig was initially infected, and in a few weeks, the other pigs living in the same sty became infected.

**Conclusions:**

Most pigs in Ibaraki Prefecture were with HEV. On the farm, most piglets were infected with HEV by the time they reached slaughter age. We confirmed that HEV infection is successively transmitted among piglets living in the same sty.

## Background

Hepatitis E virus (HEV) is a small, non-enveloped virus with a single-stranded, positive-sense RNA genome and belongs to the *Hepeviridae* family [1]. This family is divided into two genera: *Orthohepevirus* and *Piscihepevirus*. Most HEVs belong to the genus *Orthohepevirus*, which includes four species: *Orthohepevirus A* to *D* [2]. Zoonotic HEVs can be categorized into *Orthohepevirus* species *A*, which includes eight genotypes: HEV1 to 8 [3]. Genotypes 1 and 2 can only infect humans, whereas genotypes 3 and 4 can infect pigs, wild boars, humans, and other animals [4].

HEV was identified in pigs in 1997 [5]; since then, several studies have reported on pigs infected with hepatitis in various countries [6–10]. Furthermore, hepatitis infections are reportedly associated with consumption of meat products from pigs and wild boar [11–13]. Therefore, cross-species HEV transmission from animals to humans is considered the major cause of this infection in developed countries [14, 15].

HEV infection has already been reported among wild boars in Ibaraki Prefecture [16]. Although there was an HEV outbreak among humans in Ibaraki Prefecture [17], the infection situation in pigs—the most likely viral reservoir candidate—remains unclear.

Humans come into contact with pigs more readily than with wild boars; pig liver is sold in grocery stores [13]. Furthermore, pigs are suspected to be reservoirs of HEV [18, 19]; therefore, it is necessary to clarify the course of HEV infection in naturally infected pigs on farms to prevent HEV infection in pigs. Here, we investigated previous infection in pigs via antibody detection and current HEV infection via antigen detection. We clarified the history of HEV infection in pigs at slaughterhouses and the spread of infection on pig farms in Ibaraki Prefecture, Japan.

## Results

### Prevalence of anti-HEV antibody in pigs delivered to slaughterhouses

**Fig. 1:**
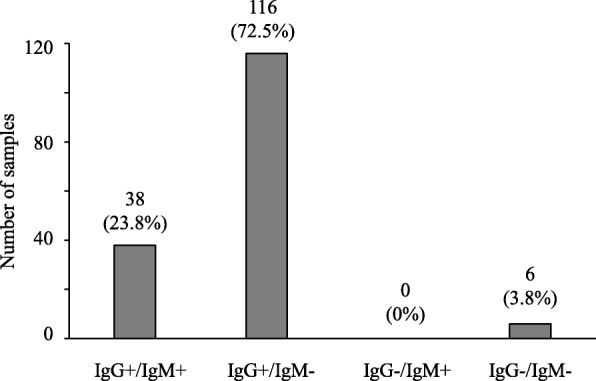
Retention ratio of the anti-HEV IgG and IgM antibodies. Anti-HEV antibodies were analyzed in 160 blood samples collected from the pig slaughterhouse. The vertical axis shows the number of samples, while the horizontal axis shows the category (IgG+ / IgM+, IgG+ / IgM-, IgG- / IgM+, and IgG- / IgM-)

Table 1 and Fig. 1 indicate the prevalence of IgG/IgM HEV antibodies in pigs from 16 commercial farms in Ibaraki Prefecture from 2015 to 2016. Of the 160 serum samples collected from the pigs, 38 (23.8%) were positive for both IgG and IgM, 116 (72.5%) were positive for IgG and negative for IgM, and 6 (3.8%) were negative for both IgG and IgM (Fig. 1). Although the IgG and IgM antibody positive rates differed for each farm, there were no farms where none of the pigs possessed the antibodies (Table 1). In contrast, *HEV* was not detected in any of the serum samples.

**Table 1:** Detection of anti-HEV antibodies in pigs at the slaughterhouse

2015				2016			
Farm	IgM	IgG		Farm	IgM	IgG	
		Negative	Positive			Negative	Positive
A	Negative	1	6	I	Negative	0	8
	Positive	0	3		Positive	0	2
B	Negative	0	7	J	Negative	0	6
	Positive	0	3		Positive	0	4
C	Negative	0	4	K	Negative	0	9
	Positive	0	6		Positive	0	1
D	Negative	0	8	L	Negative	0	7
	Positive	0	2		Positive	0	3
E	Negative	0	6	M	Negative	1	7
	Positive	0	4		Positive	0	2
F	Negative	0	9	N	Negative	0	9
	Positive	0	1		Positive	0	1
G	Negative	0	8	O	Negative	4	4
	Positive	0	2		Positive	0	2
H	Negative	0	9	P	Negative	0	9
	Positive	0	1		Positive	0	1
Total		1	79 (98.8%)	Total		5	75 (93.8%)

### Prevalence of HEV in pigs delivered to slaughterhouses

Of the 110 liver samples collected from the pigs, *HEV* was detected in only 1 liver sample (detection rate, 0.9%).

### Prevalence of HEV in breeding sows on the farm

**Fig. 2:**
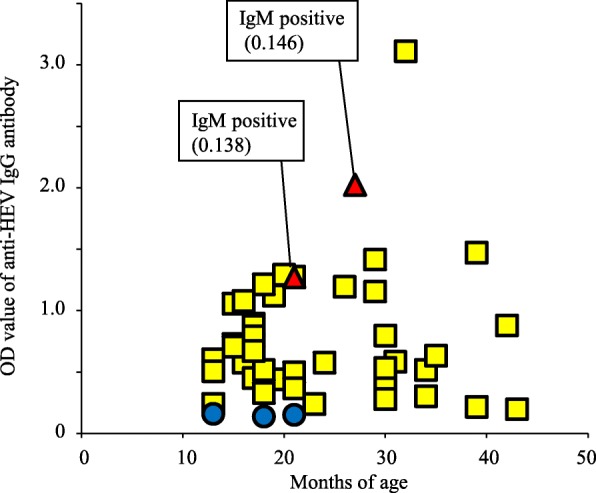
Anti-HEV IgG and IgM antibody titers and the age of breeding sows. The vertical axis shows the optical density of the anti-HEV IgG antibody titer, whereas the horizontal axis shows the age (in months) of the breeding sows in the form of a scattergram. The red triangles indicate positive IgG and IgM, yellow squares indicate positive IgG, and blue circles indicate negative IgG pigs. IgM antibody titers were noted individually

Of the 45 serum samples collected from breeding sows, 42 (93.3%) were IgG positive and 2 (4.4%) were IgM positive. Antibody titers were determined using scattergrams (Fig. 2). In addition, *HEV* was not detected in the serum samples from any breeding sow. Two serum samples were IgM positive; however, the optical density (OD) values were low and only weakly positive in both cases.

### Survey on HEV infection in piglets on the farm

**Fig. 3:**
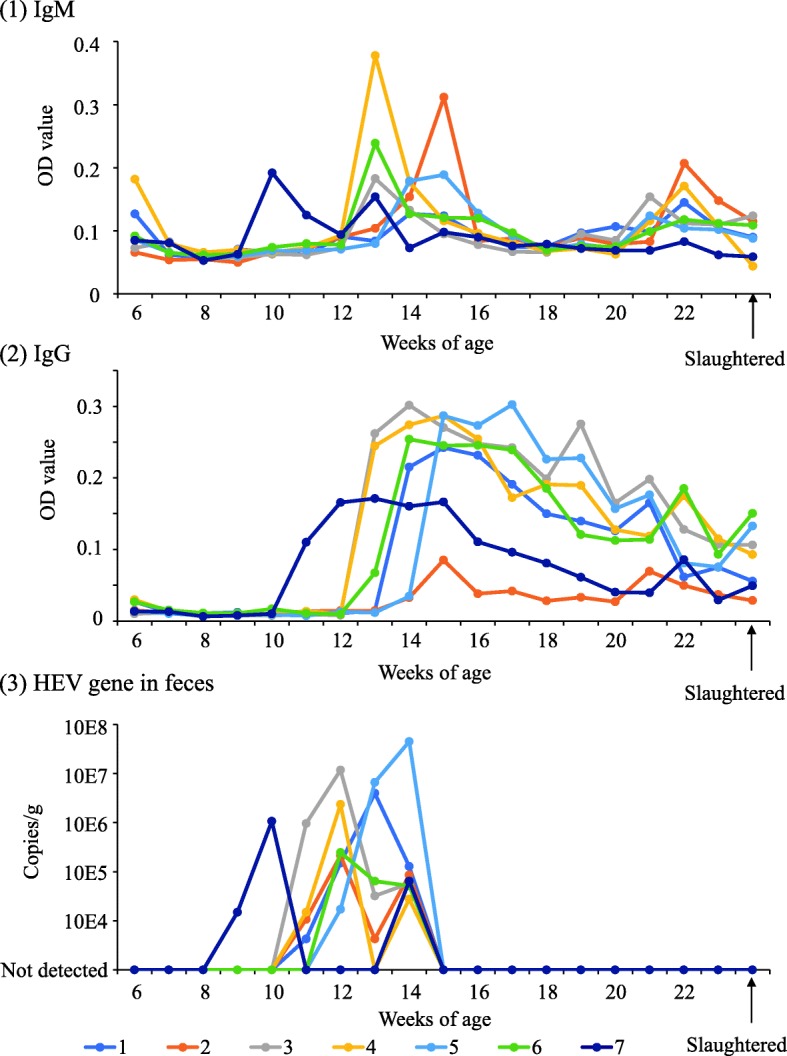
The natural infection status of HEV in piglets on the investigated farm. The piglets’ infection status is shown in chronological order. The horizontal axis shows the piglet’s age. (1) Anti-HEV IgM antibody titers. The optical density is shown on the vertical axis. (2) Anti-HEV IgG antibody titers. The optical density is shown on the vertical axis. (3) HEV gene copy numbers. The number of gene copies is shown on the vertical axis

IgM and IgG antibody transition and *HEV* detection is shown in Fig. 3. Piglet #7 excreted HEV in feces at 9–10 weeks of age, earlier than that observed for the other piglets living in the same sty. Thereafter, the concentration of IgM antibodies increased rapidly at 10 weeks of age, whereas that of IgG antibodies increased at 11 weeks of age. In contrast, four piglets (#1, 2, 3, and 4) started excreting HEV in their feces after 11 weeks of age, and the remaining two piglets (#5 and 6) started excreting HEV in their feces at 12 weeks of age. In short, after *HEV* was detected in the feces of one piglet, all other piglets were infected within 3 weeks. Although *HEV* was detected in the fecal samples for 2–4 consecutive weeks, it was not detected from 14 weeks of age until slaughter. Moreover, *HEV* was detected in only one sample (in piglet #5 at 14 weeks of age). The pigs’ internal organs were inspected after they were slaughtered. Although pneumonia, peritonitis, and pleurisy were detected, hepatitis and *HEV* were not detected in any sample.

### Sequences and phylogenetic tree analysis

**Fig. 4:**
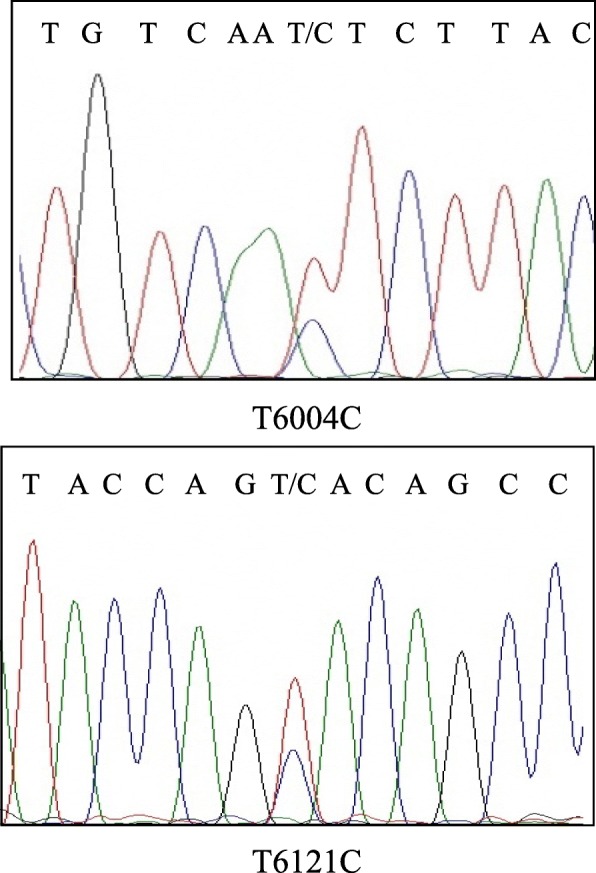
Sequence electropherogram. The two positions detected two overlapped signal peaks: T6004C and T6121C. These positions and nucleotides were the same as the two kinds of HEV strains detected in the farm. Both nucleotide mutations were synonymous in their amino acid sequences. The details have been deposited in GenBank under the accession number D10330

**Fig. 5:**
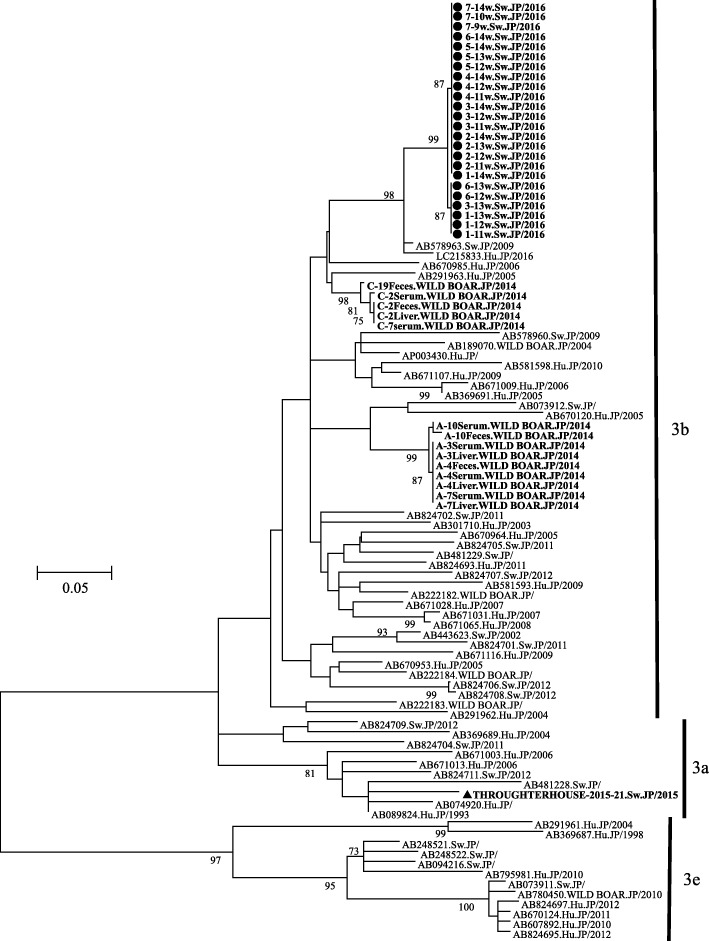
Phylogenetic tree analysis of HEV. Molecular phylogenetic tree analysis using the maximum likelihood method based on the partial sequences 336 nucleotides of ORF2 of HEV. The substitution model was GTR + gamma + Invariant. Strains collected from the farm in this study were labeled with black circles on the basis of piglet number and age in weeks. Strains collected from the slaughterhouse were labeled with black triangles. Other reference strains representatives of subtypes 3a, 3b, and 3e of the genotype 3 are shown. Numbers at branch nodes show bootstrap values with ≥70% support. Scale bar = 0.05 nucleotide substitutions per position. The strains detected in Ibaraki Prefecture were shown in bold type

As a result of phylogenetic tree analysis conducted within 336 bp of the partial segment of the capsid domain in ORF2, HEV strains on the farm were found to belong to genotype 3, subtype 3b; however, substitutions of the following two nucleotides were noted: c. 6004 T > C and c.6121 T > C (sequence position was deposited in GenBank under the accession #D10330). Both nucleotide mutations were synonymous in the amino acids contained. The 336 bp segment used for analysis is the region encoding amino acids 272–383 of the capsid protein and corresponds to the shell and middle domains [20]; the region does not encode known antigenic epitopes [21, 22]. Moreover, the HEV strains detected in piglet #2 at 13 weeks of age, in #3 at 13 weeks of age, and in #6 at 12 and 13 weeks of age showed overlapping signal peaks in the sequence data for the two nucleotides (Fig. 4). In addition, one HEV strain detected among the samples collected at the slaughterhouse belonged to genotype 3, subtype 3a, and differed from that of the strain identified on the farm (Fig. 5). HEV strains detected from piglets in this study had different sequences (Fig. 5) compared with those previously detected from wild boars in Ibaraki Prefecture [16].

## Discussion

Studies conducted in various countries worldwide have reported HEV infection in pigs and wild boars; the risk of HEV infection in humans is also well-recognized [12, 18, 23]. Here, we clarified the prevalence of HEV in pigs slaughtered in Ibaraki Prefecture and the mechanism underlying natural infection of pigs on farms.

Pigs are successively infected on farms, and the antibody positive rate increases with age [19, 24, 25]. In Japan, pigs are generally slaughtered at approximately 6 months of age; the presence of HEV at this age is negligible among pigs [10]. Here, the anti-HEV antibody IgG was detected in > 90% of the pigs, indicating that the prevalence of HEV was high in several farms. However, some pigs did not show the presence of any antibodies and the rate of infection differed for each farm. A previous study also indicated that the rate of infection varied from farm to farm [26]. HEV was detected in 1 of the 120 liver samples (0.8%) collected from the pigs at the slaughterhouse. A previous report showed that the prevalence of HEV in pig liver was 0.8–20.8% at slaughterhouses [27]. Thus, it can be inferred that HEV is present on several farms in Ibaraki Prefecture and that the prevalence of infectious pigs differs among farms and is based on the pig-raising conditions.

In contrast, the prevalence of anti-HEV IgG antibody in the breeding sows was as high as that in pigs of slaughtering age. In short, it is speculated that the sows did not infect piglets with HEV because they had been previously infected and possessed antibodies. A previous report showed that in pigs, the antibody positive rate increases with age [9]. Furthermore, most pigs of slaughtering age are already infected with HEV on the farm and their anti-HEV IgG antibody titer is maintained for several years [28]. Therefore, it was speculated that breeding sows did not amplify HEV and did not transmit the virus to their piglets.

In this study, one piglet was infected with HEV at an age younger than that of the other six piglets in the same sty. The former piglet excreted HEV in its feces at 9–10 weeks of age. Thereafter, the other six piglets excreted HEV in their feces at 11–14 weeks of age. Andraud et al. demonstrated that the mean period from contact of infection to fecal HEV excretion was 7.1 days, consistent with a previous report [19, 29]. In contrast, serum sample analysis showed positive detection only in piglet #5 at 14 weeks of age. A previous report showed that HEV was difficult to detect from serum samples [30]. Fluctuations in antibody titers and changes in virus excretion in piglets reportedly depend on whether the sows are infected with HEV [31]. In the present study, piglets from the same sow were used and the dynamics of antibody and virus release were considered to be almost the same. Furthermore, a previous report showed that HEV infection does not occur unless an adequate viral load is ingested [32]. Nevertheless, in this study, there were no opportunities for pigs living in one sty to come into close contact with other pigs in another sty or in a separate herd. It is unlikely that a sufficient amount of virus for infection was suddenly exposed to all piglets. From these facts, it was suspected that one piglet infected all the other piglets living in the same sty between 9 and 14 weeks of age. However, because this study was aimed at monitoring natural infections, we could not completely exclude the possibility of separate or multiple introductions of different HEV viruses into the piglets and the initial intruded cause could not be clarified.

Phylogenetic tree analysis revealed that HEV strains identified on the same farm were extremely closely related; however, strain identified from another farm at the slaughterhouse was of a different subtype. Previous reports have shown that the HEVs detected on different farms differed from each other in genotype and gene sequence [8, 19, 33], whereas those detected on the same farm were similar [34]. These facts suggest that similar strains spread on the same farm. Moreover, HEV sequence electropherograms from piglets #2 (at 13 weeks of age), #3 (at 13 weeks of age), and #6 (at 12–13 weeks of age) had overlapping peaks for the two nucleotides; these piglets were probably co-infected with two different HEVs detected on the farm.

## Conclusions

This study revealed the dynamics of HEV in naturally infected farm pigs and the history of infection in slaughtered pigs in Ibaraki Prefecture. Most pigs of slaughtering age were infected with HEV, retained antibodies and excreted them; this common mechanism suggests that HEV is present on most farms in Ibaraki Prefecture. Moreover, breeding sows possess antibodies and their piglets become infected at approximately 3 months of age. We found that all piglets are not infected at once; rather, one piglet is infected first and it then transmits the virus to the other piglets living in the same sty. We also suspected that the piglets may have been co-infected with two different HEVs detected on the farm.

## Methods

### Materials

To investigate the actual status of HEV infection in Ibaraki Prefecture, we collected samples from pigs at slaughterhouses. Blood samples were collected from 160 pigs sent to the slaughterhouse between August 2015 and October 2016. Of these, 10 were selected from 8 farms, such that 80 pig serum samples were collected each year. In contrast, a total of 110 pig livers that tested positive for hepatitis were collected between September 2015 and March 2016 from individual animals other than those from whom the blood samples were collected.

**Fig. 6:**
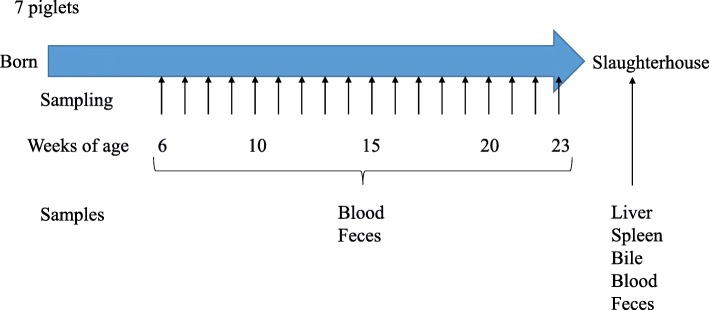
Schedule of piglet sampling on the farm. After the seven piglets were born, they were raised in the same sty on the farm. We collected blood and feces (rectal swabs) every week from 43 days (6 weeks) to 166 days (23 weeks) of age. These piglets were finally slaughtered, and we collected the liver, spleen, bile, blood, and fecal samples from the slaughterhouse

Next, we collected 45 blood samples from all breeding sows on a single farm in November 2015. Moreover, blood and fecal (rectal swab) samples were collected every week from 43 to 166 days of age in seven Landrace piglets born from the same sow on May 8, 2016 and raised in the same sty on the same farm. The samples were collected 18 times on the farm. The liver, spleen, bile, blood, and fecal samples were collected at a slaughterhouse on October 21, 2016 (Fig. 6).

### Sample preparation

Blood and bile samples were centrifuged for 10 min at 1500 *g* and the serum and supernatant, respectively, were collected for analysis. Feces (rectal swabs) were diluted with PBS to generate a 10% suspension and centrifuged for 10 min at 10,000 *g.* The supernatant was collected for analysis. Liver and spleen sample suspensions were prepared in PBS using a multi-beads shocker (YASUI KIKAI) [35] and centrifuged for 10 min at 10,000 g; the supernatant was then collected for analysis.

### Detecting HEV RNA and anti-HEV antibody

HEV nucleic acid was extracted from the samples using the QIAamp Viral RNA Mini Kit (QIAGEN), treated with 10 U of DNaseI (Takara), and reverse transcribed using the PrimeScript RT Master Mix (Takara). The resulting cDNA samples were analyzed using quantitative PCR [36]. The sera were used for detecting anti-HEV IgM and IgG antibodies by ELISA [37, 38].

### Sequence analysis

For *HEV* classification genotyping, 336 bp of the partial segment of the capsid domain in ORF2 was amplified using the PrimeScript II High Fidelity One Step RT-PCR kit (Takara) and subjected to sequence determination using the BigDye Terminator v3.1 Cycle Sequencing Kit (Thermo Fisher Scientific) [39]. A phylogenetic tree was constructed using the KAKUSAN4 program [40] and MEGA6 software [41]. The strains were subjected to phylogenetic analysis at ≥70% bootstrap support (1000 iterations). The strains detected in Ibaraki Prefecture; in this study and in a previous study were showed in bold type [16].

## Abbreviations

HEV: Hepatitis E virus
OD: Optical density
